# Proteomic Analysis of *Bifidobacterium animalis* AR668 and AR668-R1 Under Aerobic Culture

**DOI:** 10.3390/foods14101766

**Published:** 2025-05-16

**Authors:** Yaping Liu, Xiaoxiao Zhao, Miao Yang, Xin Song, Guangqiang Wang, Yongjun Xia, Liang Zhao, Zhiqiang Xiong, Lianzhong Ai

**Affiliations:** 1 Shanghai Engineering Research Center of Food Microbiology, School of Health Science and Engineering, University of Shanghai for Science and Technology, Shanghai 200093, China; lyplyp_lll@163.com (Y.L.); 18595398556@163.com (X.Z.); yang3shuihehe@163.com (M.Y.); daohongxuan@126.com (X.S.); 1015wanggq@163.com (G.W.); dreamup@126.com (Y.X.); ailianzhong1@126.com (L.A.); 2 College of Food Science and Nutritional Engineering, China Agricultural University, Beijing 100083, China; lzhao@cau.edu.cn; 3 School of Agriculture and Biology, Shanghai Jiao Tong University, Shanghai 200240, China

**Keywords:** *Bifidobacterium animalis*, oxygen-tolerant, proteomic analysis

## Abstract

*Bifidobacterium animalis* is a widely used probiotic with significant health benefits, but its application is limited by oxygen sensitivity. Our laboratory previously developed an oxygen-tolerant *B. animalis* AR668-R1 using adaptive laboratory evolution under aerobic culture, but the molecular mechanism remains unclear. In this work, compared to the wild-type parental strain *B. animalis* AR668, 212 upregulated and 390 downregulated proteins were identified in AR668-R1 under aerobic conditions through comparative proteomic analysis. Enrichment analysis of the differentially expressed proteins between AR668 and AR668-R1 identified the potential oxygen-tolerant related pathways, including the translation process, transmembrane transport system, and carbohydrate metabolism. Furthermore, five potential oxygen-tolerance proteins (DapE, Mth2, MutT, Eno, and MsrAB) were validated by RT-qPCR that may contribute to the aerobic growth of AR668-R1. Through gene overexpression validation, Mth2 (7,8-dihydro-8-oxoguanine triphosphatase) was found to enhance the growth of AR668-R1 by 19.8% compared to the empty plasmid control under aerobic conditions. Our finding provides valuable insights into the oxygen-tolerant mechanisms of *B. animalis* at the protein level.

## 1. Introduction

*Bifidobacterium animalis*, a Gram-positive, anaerobic bacterium, is widely recognized for beneficial effects on human health, particularly the regulation of the gut microbiota. This probiotic strain is characterized by its ability to ferment carbohydrates producing lactic acid and acetic acid, which contributes to the maintenance of a healthy intestinal environment [1]. The physiological properties of *B. animalis* include the ability to adhere to intestinal epithelial cells, modulate immune responses, and produce antimicrobial compounds that inhibit the growth of pathogenic bacteria [2]. These characteristics make *B. animalis* a valuable component of functional foods and dietary supplements aimed at promoting gut health. In the food industry, *B. animalis* has been extensively utilized in the production of fermented dairy products such as yogurt, cheese, and kefir [3], which enhance food nutritional value and sensory properties, such as texture and flavor [4]. Additionally, *B. animalis* has been incorporated into non-dairy products, including infant formula, cereals, and beverages, to provide probiotic benefits to a wider range of consumers [5]. Despite the widespread application, a significant limitation of *B. animalis* is sensitivity to oxygen, which restricts viability and functionality in aerobic environments. This oxygen sensitivity poses challenges for the storage, distribution, and incorporation of *B. animalis* into non-fermented foods and supplements, where exposure to oxygen is inevitable.

The oxidative stress response in model organisms such as *E. coli* is well characterized, depending primarily on the OxyR/SoxRS regulatory systems and classical reactive oxygen species (ROS)-scavenging enzymes including catalase and superoxide dismutase [6]. However, *Bifidobacterium* species are notably lacking in these conserved oxidative defense mechanisms [7,8]. Recent studies revealed that *B. breve* UCC2003 employs an alternative stress response strategy mediated by a coordinated regulatory network. This network harbors ClgR (stress-responsive transcriptional regulator), LexA (the DNA damage response regulator), and HrcA and HspR (heat shock protein regulators) [9]. For hydrogen peroxide detoxification, *B. bifidum* primarily relies on alkyl hydroperoxide reductase (Ahp) and the thioredoxin reductase-AhpC (TrxR-AhpC) antioxidant system [10]. Furthermore, oxygen tolerance is facilitated by NADH oxidase (NOX) and HemN-like oxygen reductase activity [11]. Notably, *B. longum* LTBL16 could enhance oxidative resistance through the upregulation of peroxidases and the activation of the NOX-dependent FoxO signaling pathway [12]. These findings collectively demonstrated that *Bifidobacterium* species develop diverse and specialized mechanisms to mitigate oxidative stress.

However, despite these intrinsic defense systems, most bifidobacteria remain highly sensitive to oxygen exposure, which severely limits their viability during industrial production and food applications where aerobic conditions are unavoidable. To address this limitation, adaptive laboratory evolution (ALE) strategies for selecting oxygen-tolerant strains have been extensively employed to engineer starter cultures with enhanced robustness for industrial food fermentation processes [13]. Wild-type parental strain *B. animalis* AR668 was isolated from infant feces [14]. The oxygen-tolerant strain *B. animalis* AR668-R1 was obtained after the ALE adaptation of AR668 [15] (Figure S1). We previously employed genomic and phenotypic analyses to elucidate the potential oxygen tolerance mechanism in *B. animalis* AR668-R1, revealing that membrane proteins and ABC transporter are involved in conferring oxygen tolerance of *B. animalis*. However, there is still a need for a comprehensive understanding of the molecular changes that occur during oxygen adaptation [16]. Here, this study aimed to identify potential oxygen-tolerant proteins in *B. animalis* AR668-R1 using proteomic analysis, which provides valuable insights into the oxygen-tolerant mechanism by which bifidobacteria cope with oxidative stress at the protein level.

## 2. Materials and Methods

### 2.1. Strain, Media, and Culture Condition

The bacterial strains and plasmids are listed in Table 1. *B. animalis* AR668 and AR668-R1 were cultivated in a BS medium (Catalog #HB0394-1, Hope Bio, Qingdao, China) at 37 °C under anaerobic conditions without shaking or aerobic conditions with shaking at 100 rpm.

### 2.2. Hydrogen Peroxide Tolerance and Cell Growth of AR668 and AR668-R1

The overnight culture of AR668 or AR668-R1 (OD_600_ = 1.0–1.2) was adjusted to achieve an OD_600_ of 0.60 ± 0.05. The bacterial suspension was then added to sterile BS broth containing hydrogen peroxide (H_2_O_2_, Catalog #H1009, Sigma-Aldrich, St. Louis, MO, USA) at concentrations ranging from 3.5 mM to 10 mM, followed by incubation for 3 h. The control group consists of BS broth without H_2_O_2_. After appropriate dilution, the survival rate of AR668 or AR668-R1 under H_2_O_2_ stress was calculated using the colony counting method on BS agar plates. Each group was repeated three times.

An overnight culture of AR668 or AR668-R1 was inoculated at 5.0% into 100 mL of the BS medium under aerobic conditions for 24 h. The absorbance at 600 nm was measured every 2 h, with three replicates each time. The colony-forming units per milliliter (CFU/mL) of AR668 or AR668-R1 was obtained by plating 10 μL stationary phase cultures on the BS medium at 37 °C for 24 h under anaerobic conditions by 10-fold serial dilutions.

### 2.3. Protein Sample Preparation, Sequencing, and Enrichment Analysis

The strains AR668-R1 and AR668 were grown in the BS liquid medium with agitation at 100 rpm until reaching the early logarithmic phase. The cells were harvested by centrifugation (4 °C, 4000× *g*, 10 min), flash-frozen in liquid nitrogen for 10 min, and lysed in a protein lysis buffer (Catalog #B14001, Bimake, TX, USA) containing 8 M urea, 1.0% SDS, and protease inhibitors. Homogenization was performed using a high-throughput tissue grinder (3 cycles, 40 s each), followed by incubation on ice for 30 min with brief vortexing every 5 min. After centrifugation (16,000× *g*, 4 °C, 30 min), the supernatant was collected. Protein integrity was verified by an SDS-PAGE analysis, followed by concentration quantification using a BCA Protein Assay Kit (Catalog #23227, Thermo Fisher Scientific, Waltham, MA, USA). For digestion, 100 μg of protein was reduced with 10 mM Tris (2-carboxyethyl)phosphine (TCEP, Catalog #20490, Thermo Fisher Scientific, MA, USA) at 37 °C for 60 min, alkylated with 40 mM iodoacetamide (IAM, Catalog #I6125, Sigma-Aldrich, St. Louis, MO, USA) in the dark for 40 min, and digested overnight at 37 °C using trypsin (Catalog #V5111, Promega, Madison, WI, USA) at a 1:50 enzyme–protein ratio. After protein digestion, peptides were desalted using HLB (Hydrophilic-Lipophilic Balanced) cartridges, dried, and reconstituted in 0.1% trifluoroacetic acid (TFA, Catalog #PI85183, Thermo Fisher Scientific, MA, USA).

The peptides were resuspended in a spectrometry loading buffer (2.0% ACN, 0.1% formic acid) with iRT peptides for retention time calibration. An LC-MS/MS analysis was conducted on an EASY nLC-1200 system coupled to a Q Exactive HF-X mass spectrometer (Thermo Fisher Scientific, MA, USA) at Majorbio Bio-Pharm Technology Co., Ltd. (Shanghai, China). The data-independent acquisition (DIA) mode was employed, alternating between full-scan MS and MS/MS. The C18 reversed-phase column (75 μm × 25 cm, Thermo, USA) was equilibrated with solvent A (2% acetonitrile with 0.1% formic acid) and solvent B (80% acetonitrile with 0.1% formic acid) at a flow rate of 300 nL/min. The peptides were eluted using the following gradient: 0–70 min, 5–23% B; 70–90 min, 23–29% B; 90–100 min, 29–38% B; 100–102 min, 38–48% B; 102–103 min, 48–100% B; and 103–120 min, 100% B (maintained).

The raw data were processed using Spectronaut™ (v14) with iRT-based retention time adjustment, in which the database search software automatically performs internal normalization during differential protein quantification. The quantification relied on 6 peptides per protein and 3 daughter ions per peptide, excluding shared/modified peptides. Proteomic data were analyzed on the Majorbio Cloud platform. Differential protein expression was determined using an R-based *t*-test (with thresholds set at fold change > 1.20 or <0.83, *p* < 0.05). Functional annotation and enrichment of differentially expressed proteins (DEPs) were performed via GO (http://geneontology.org/, accessed on 10 March 2025) and KEGG (http://www.genome.jp/kegg/, accessed on 10 March 2025) databases.

### 2.4. RNA Preparations and Gene Expression Analysis by Reverse Transcription Quantitative Polymerase Chain Reaction (RT-qPCR)

Total RNA was isolated using a total RNA extraction reagent (Catalog #B511311, Sangon Biotech, Shanghai, China). Due to the thick cell wall of *B. animalis*, lysozyme was used for 1 h pretreatment to extract high-quality RNA [18]. The integrity and quantity of RNA were determined by agarose gel electrophoresis and NanoDrop 2000 spectrophotometry (Thermo Fisher Scientific, MA, USA), respectively. Complementary DNA (cDNA) was prepared by the PrimeScriptRT reagent kit (Catalog #RR037A, Takara Bio Inc., Shiga, Japan) and used for RT-qPCR. cDNA synthesis was performed as follows: 1 pg^−1^ μg RNA was mixed with 4 μL 4× *g* DNA wiper Mix in a total volume of 16 μL RNase-free dH_2_O (42 °C, 2 min), followed by the addition of 4 μL 5× HiScript III qRT SuperMix (37 °C, 15 min) and enzyme inactivation (85 °C, 5 s). cDNA concentration was measured by Nanodrop 2000 at A260. RT-qPCR was performed using SYBR Premix Ex Taq (Catalog #DRR420A, Takara Bio Inc., Shiga, Japan), with each reaction containing 10 μL of 2 × SYBR Green Mix, 0.5 μL of cDNA, 0.5 μL of each primer (20 μmol/L), and 8.5 μL of ddH_2_O, gently mixed in a PCR tube. The PCR reactions were carried out as follows: initial denaturation at 95 °C for 30 s, followed by 40 cycles of denaturation at 95 °C for 5 s and annealing/extension at 60 °C for 30 s, with a final extension at 72 °C for 5 min. The gene-specific qPCR primers are listed in the Supplementary Information (Table S2). The reference gene *rplD* verified in previous experiments was selected as a control [7]. The qPCR reactions were performed in 96-well (LightCycle 8-tube strips), and three repetitions were made for each sample.

### 2.5. Plasmid Construction

The gene sequences and primers are listed in the Supplementary Information (Tables S1 and S2). The DNA fragments of *gene0321*, *gene0618*, *gene0924*, *gene1072*, and *gene1102* were amplified from the *B. animalis* genome by PCR using primers and inserted into the *E. coli*-*B. animalis* shuttle plasmid pAM1-ldh2 with digestion by *Hin*dIII and *Xba*I using the ClonExpress one-step cloning kit (Catalog #C113, Vazyme Biotech Co. Ltd., Nanjing, China). The overexpression plasmids were validated by PCR (Figure S2). All the plasmids were constructed in *E. coli* Top10, cultured in Luria–Bertani (LB) broth supplemented with 100 μg/mL ampicillin (Amp) at 37 °C overnight with shaking (200 rpm). The amplified plasmids were electroporated into *B. animalis*, and cultured in BS medium supplemented with 5 μg/mL erythromycin (Em) at 37 °C under anaerobic conditions without shaking. Empty plasmid ldh2-pAM1 was used as a negative control.

### 2.6. Statistical Analysis

The results were statistically analyzed by one-way analysis of variance using the statistical software package SPSS 26.0. The statistical degree of significance was set at * *p* < 0.05, ** *p* < 0.01, and *** *p* < 0.001.

## 3. Results and Discussion

### 3.1. Hydrogen Peroxide Tolerance and Cell Growth of AR668-R1 and AR668

**Figure 1:**
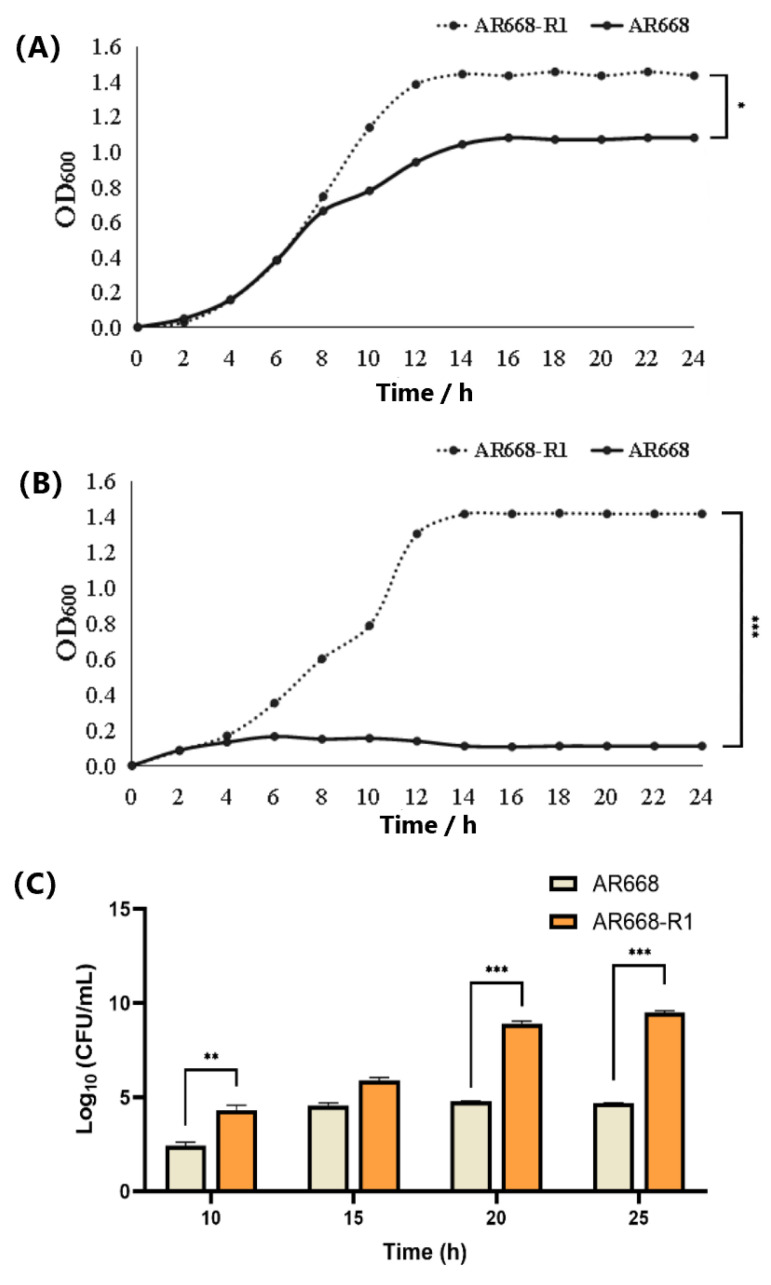
The growth curves of *B. animalis* AR668 and AR668-R1 under anaerobic (**A**) and aerobic (**B**) conditions. Determination of the viable cell counts of AR668 and AR668-R1 cultured under an aerobic environment (**C**). * *p* < 0.05, ** *p* < 0.01 and *** *p* < 0.001 by one-way analysis of variance.

The maximum lethal concentration of H_2_O_2_ for AR668 and AR668-R1 was determined using the plate counting method (Table S3). It was found that AR668 completely died at 6.0 mM H_2_O_2_, while AR668-R1 retained viability (9.7% survival) even at 8.5 mM H_2_O_2_. Under anaerobic conditions, both AR668 and AR668-R1 grew normally (Figure 1A). AR668-R1 reached a maximum OD_600_ of 1.46, which was significantly higher than that of AR668 (1.08, *p* < 0.05). The anaerobic growth improvement observed in AR668-R1 may result in ALE-driven metabolic reorganization, which has been demonstrated to provide oxidative stress tolerance in *S. cerevisiae* [19]. Under aerobic conditions, the maximum growth OD_600_ of AR668-R1 reached 1.44, corresponding to 3.09 × 10^9^ CFU/mL (Figure 1B,C). This indicates that AR668-R1 could grow normally in the aerobic environment. In contrast, the growth of AR668 was severely inhibited under aerobic conditions. Therefore, the oxygen tolerance of AR668-R1 showed a significant improvement through ALE adaptation compared to AR668.

### 3.2. Functional Enrichment Significance Analysis

**Figure 2:**
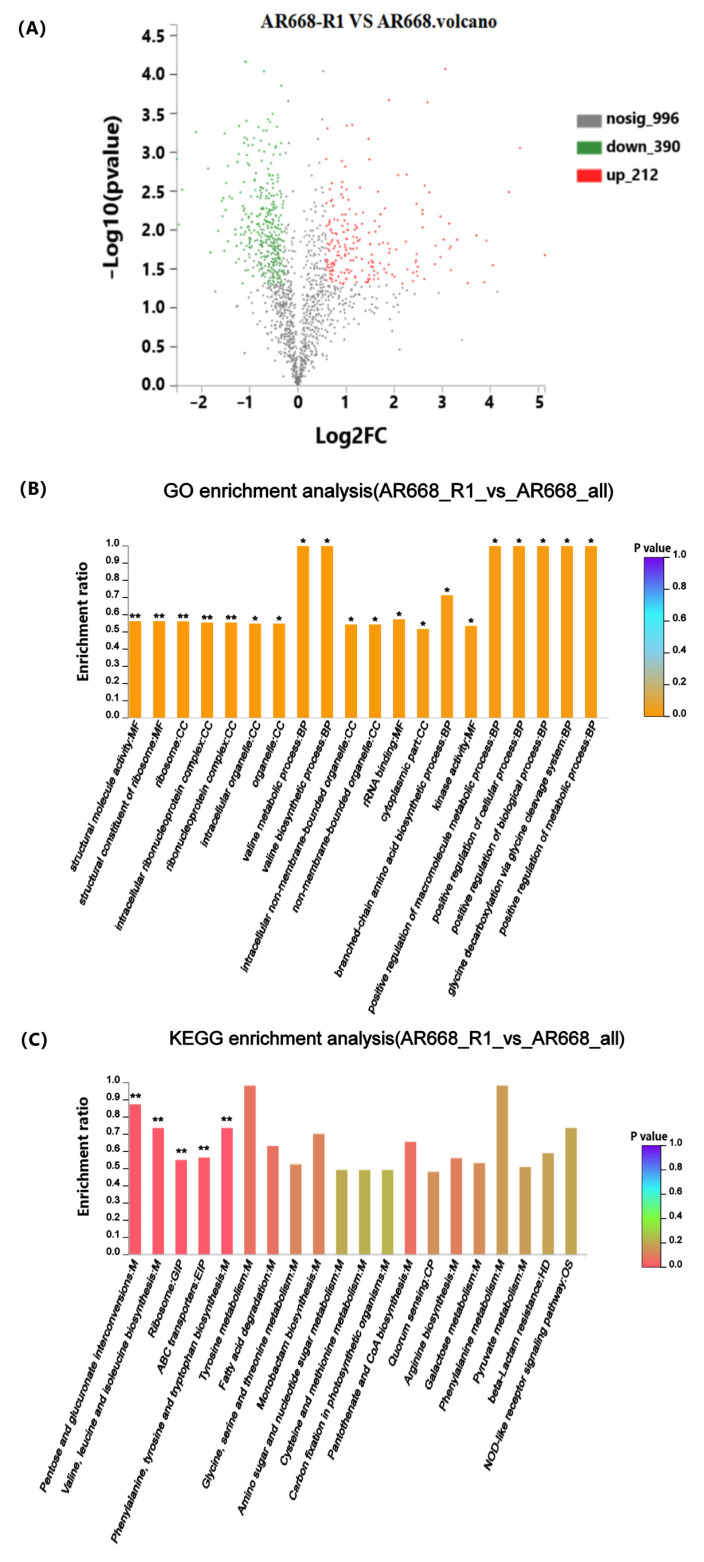
Functional enrichment significance analysis. Volcano plot of differentially expressed genes between *B. animalis* AR668 and AR668-R1 under anaerobic conditions. Each dot represents an individual gene. The red dots indicate significantly upregulated genes, the green dots represent significantly downregulated genes, and the gray dots denote genes without significant differences (**A**). GO (**B**) and KEGG (**C**) functional enrichment significance analysis of differentially expressed proteins in *B. animalis* AR668 and AR668-R1 under aerobic conditions. The color scale indicates enrichment significance, with increasingly orange/red hues representing more significant enrichment (* *p* < 0.05 and ** *p* < 0.01).

To understand the oxygen tolerance mechanism of AR668-R1, the protein expression levels of AR668-R1 and AR668 under aerobic conditions were determined by proteomic analysis, followed by functional annotation and pathway analysis using GO and KEGG. A total of 1598 proteins were detected in AR668 and AR668-R1, with 212 upregulated proteins and 390 downregulated proteins in AR668-R1 using AR668 as the control (Figure 2A). The top five significant (*p* < 0.01) GO functional enrichment results were all related to ribosomes, which function to synthesize polypeptide chains from amino acids according to mRNA instructions (Figure 2B). It indicated that the cultivation of *B. animalis* under an aerobic environment primarily affects the translation process, including protein synthesis and amino acid metabolism. In addition, the GO analysis revealed significant enrichment (*p* < 0.05) in valine metabolism and synthesis, rRNA binding, branched-chain amino acid synthesis, kinase activity, glycine cleavage, and the positive regulation of metabolic processes, which were potentially associated with oxygen tolerance in *B. animalis*. Similarly, the KEGG pathway analysis showed significant (*p* < 0.01) enriched pathways including pentose and glucuronate interconversions; valine, leucine, and isoleucine biosynthesis; ribosomes; and ABC transport systems (Figure 2C). It suggested that in addition to protein synthesis, the pathways related to carbohydrate metabolism and transmembrane transport systems are also important to oxygen tolerance.

### 3.3. Analysis of Differential Expressed Proteins

**Figure 3:**
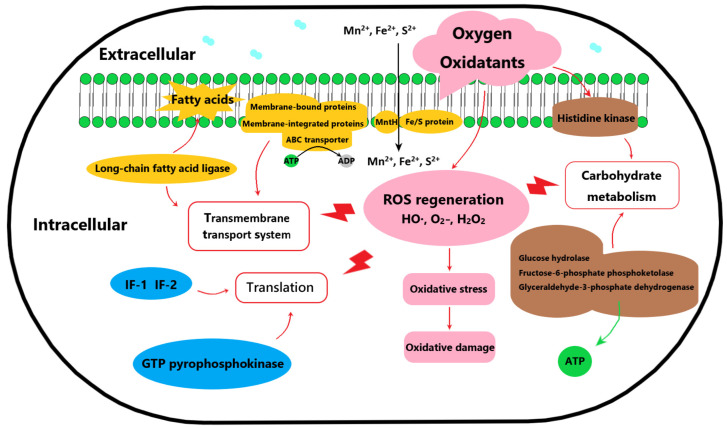
A summary of the involved pathways of oxygen tolerance in *B. animalis* AR668-R1.

Protein expression with significant differences (*p* < 0.01) were analyzed in the proteomic data (Table 2). Compared to AR668, 37 upregulated (FC > 2.0) and 38 downregulated (FC < 0.5) proteins were identified in AR668-R1. Based on the function of the differential proteins, a summary of the involved pathways associated with oxygen tolerance was found in AR668-R1 (Figure 3). For example, the ribosome serves as the exclusive site for protein biosynthesis, and proteins associated with ribosome modification and translation, including small subunit ribosomal RNA methyltransferase G (24.48-fold), 30 S ribosomal protein S6 (8.34-fold), and 50 S ribosomal proteins L24 and L33 (3.23-fold and 3.17-fold, respectively), were significantly upregulated in AR668-R1. Translation initiation factors IF-1 and IF-2 were also upregulated, suggesting the enhanced translation efficiency in AR668-R1. During the translation process, the elongation factor (EF) promotes peptide chain elongation, while the RelE factor cleaves messenger RNA at specific ribosomal sites to terminate translation. Therefore, the upregulation of RelE inhibits global protein synthesis, which leads to persistence and enhances aerobic survival capacity [20]. Notably, the GTP pyrophosphokinase protein enhances RelE activity, thereby significantly reducing translation efficiency [21]. Collectively, EF, IF−1, and IF-2 were upregulated 2.31- to 5.52-fold, while the GTP pyrophosphate kinase protein was downregulated by 4.34-fold. These differential expressions demonstrated faster translation speed and higher precision in protein synthesis under aerobic culture, which were directly associated with oxygen tolerance in *B. animalis*.

The cell membrane fatty acid composition is critical for stress resistance in *Bifidobacterium* [22]. In AR668-R1, long-chain fatty acid ligase and membrane-bound proteins were obviously downregulated, while ABC transporter proteins, implicated in substrate transport and oxygen tolerance [23], were upregulated (5.52-fold, 4.77-fold, and 3.03-fold for putative transporter protein, conserved membrane protein, and ABC transporter permease, respectively). Additionally, AR668-R1 exhibited enhanced carbohydrate metabolism, with a 2.8-fold upregulation of glyceraldehyde-3-phosphate dehydrogenase, a key enzyme in glycolysis [24], leading to elevated NADH/NADPH generation and ATP production under oxygen stress [25]. The two-component system in AR668-R1 was also more active, evidenced by a 2.44-fold upregulation of histidine kinase, enabling rapid environmental signal response and stress adaptation. As revealed by the proteomic analysis, the potential oxygen tolerance mechanism of AR668-R1 involves enhanced translation efficiency, improved transmembrane transport for membrane repair, and accelerated carbohydrate metabolism, which constitute intrinsic defense systems against oxidative stress (Figure 3). The coordinated extracellular protection and intracellular metabolic and translational adaptations enable AR668-R1 to maintain cellular integrity and function under aerobic conditions.

### 3.4. Validation of Potential Oxygen-Tolerance Proteins of B. animalis

According to the above proteomic analysis of differentially expressed proteins and our previous genomic analysis [15], we excluded certain proteins based on their functional annotation and literature evidence in metabolic pathways, including ribosome biogenesis-related proteins, non-oxygen-tolerant related proteins (e.g., cold-shock protein), and uncharacterized proteins. Notably, the upregulation of ribosomal proteins and translation factors in AR668-R1 indicated an enhanced translational capacity, which may contribute to oxygen tolerance through the rapid synthesis of stress-response proteins during oxidative challenge. This observation was supported by a previous report on *E. coli*, in which oxidative stress-resistant mutants increased the expression of translation machinery components to maintain proteostasis under ROS exposure [26]. The remaining candidates were evaluated through their functional contributions to essential oxygen-tolerance mechanisms, including membrane integrity maintenance demonstrated by membrane-bound transporters, oxidative DNA damage repair involving 8-oxo-dGTPase, energy metabolism through glycolytic enzymes, and redox homeostasis maintained by methionine sulfoxide reductases (Table 3). We further selected the following five proteins for experimental validation to confirm their roles that may be involved in the oxygen tolerance of AR668-R1. DapE (membrane protein) and Mth2 (7,8-dihydro-8-oxoguanine-triphosphatase) were selected based on the function in maintaining membrane stability under oxidative stress, as demonstrated in studies of oxygen stress responses in lactic acid bacteria [27]. MutT (DNA mismatch repair protein) was prioritized for its function as a crucial regulator in mitigating DNA damage during oxidative stress, reducing stress-induced oxidative damage by enhancing gene expression levels [11]. Eno (enolase) was included due to its regulatory function in diverse stress responses [28], while MsrAB (peptide methionine sulfoxide reductase) was chosen as a key component of the ModRS-regulated oxidative stress defense system [29].

**Figure 4:**
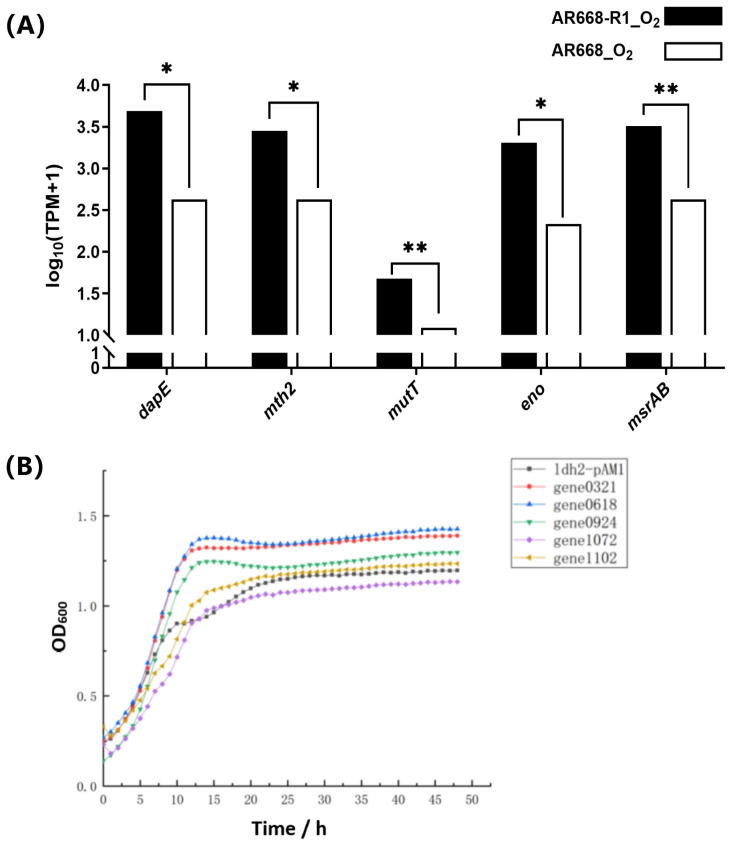
Validation of key proteins. The mRNA expression levels of potential oxygen-tolerance proteins by RT-qCPR using *rplD* as the control in *B. animalis* AR668 and AR668-R1 under aerobic conditions. * *p* < 0.05 and ** *p* < 0.01 by one-way analysis of variance (**A**). Aerobic growth curve of recombinant *B. animalis* AR668-R1 (**B**).

The gene expression of these five potential oxygen-tolerance proteins was analyzed using RT-qPCR in *B. animalis* AR668 and AR668-R1 under aerobic conditions (Figure 4A). The expression levels of *gene0321* (*dapE*), *gene0618* (*mth2*), *gene0924* (*mutT*), *gene1072* (*eno*), and *gene1102* (*msrAB*) were significantly (*p*  <  0.05) upregulated, which indirectly indicated the correctness of our proteome data. To further validate these five potential oxygen-tolerant proteins, we overexpressed their corresponding genes in *B. animalis* AR668-R1 using the ldh2 promoter. The RT-qPCR analysis showed that all the target mRNAs were upregulated, with transcript levels increasing >3-fold compared to the control (Figure S3). Under aerobic conditions, compared to the control strain, the overexpression of *gene0321*, *gene0618*, and *gene0924* resulted in better growth (Figure 4B). Specifically, the OD_600_ of the strain overexpressing *gene0321* and *gene0618* were 17.6% and 19.8% higher than the control during the stationary phase, respectively. It confirmed that the DapE and Mth2 proteins are associated with the oxygen tolerance of *B. animalis*, providing valuable insights into the oxygen-tolerant mechanisms of *B. animalis* at the protein level. By analyzing the oxygen tolerance mechanism of the aerotolerant *B. animalis* AR668-R1 strain, this study provides a theoretical foundation for developing oxygen-resistant *Bifidobacterium*. Additionally, the inherent oxygen sensitivity of *B. animalis* has previously limited its applications in food products. Its wider application in fermented foods will be made possible by clarifying its oxygen tolerance mechanism. Furthermore, this insight can serve as a valuable reference for the genetic engineering of other oxygen-resistant strains, expanding the use of probiotics in a broader range of applications.

## 4. Conclusions

*Bifidobacterium animalis* AR668-R1, developed by ALE, exhibited high oxygen tolerance in comparison to the parental strain AR668. Comparative proteomic analysis identified 212 upregulated and 390 downregulated proteins in AR668-R1, revealing that the potential oxygen tolerance mechanism involves extracellular membrane components and intracellular transport systems to mitigate oxidative damage. Five potential oxygen-tolerance proteins (DapE, Mth2, MutT, Eno, and MsrAB) were validated, with Mth2 overexpression significantly improving growth by 19.8%. These findings provide valuable insights into the molecular basis of oxygen tolerance in *B. animalis*.

## Figures and Tables

**Table 1:** Strains and plasmids.

Strains/Plasmids	Descriptions	References
Strains		
*Escherichia coli* Top10	Host for cloning	Our laboratory
*B. animalis* AR668	Wild-type, isolated from infant feces	Our laboratory
*B. animalis* AR668-R1	High oxygen-tolerant, obtained through ALE using AR668 as the parental strain	Our laboratory
Plasmids		
pAM1-ldh2	Em^r^ (erythromycin resistance), Amp^r^ (ampicillin resistance); pAM1 derivative for promoter P_ldh2_ replacement	[17]
pLYP0321	Em^r^, Amp^r^; pAM1-ldh2 derivative containing the *gene0321*	This work
pLYP0618	Em^r^, Amp^r^; pAM1-ldh2 derivative containing the *gene0618*	This work
pLYP0924	Em^r^, Amp^r^; pAM1-ldh2 derivative containing the *gene0924*	This work
pLYP1072	Em^r^, Amp^r^; pAM1-ldh2 derivative containing the *gene1072*	This work
pLYP1102	Em^r^, Amp^r^; pAM1-ldh2 derivative containing the *gene1102*	This work

**Table 2:** Differentially expressed proteins in *B. animalis* AR668 and AR668-R1 under aerobic conditions. Fold change (FC) represented the ratio of protein expression between the AR668-R1 and AR668 strains.

The ID in Database	Symbol	Function	Differential Regulation	FC (AR668-R1/AR668)
A0A2M9HL62	rsmG	Ribosomal RNA small subunit methyltransferase G	up	24.48
A0A0L7CYV1	cspA	Cold-shock protein	up	20.85
D3R4Y3	msrAB	peptide methionine sulfoxide reductase MsrA/MsrB	up	8.82
A0A087BER2	rpsF	30S ribosomal protein S6	up	8.34
A0A315RYH0	TMEM175	DUF1211 domain-containing protein	up	7.78
D3R6P3	-	Capsular polysaccharide synthesis protein	up	6.63
D3R6Y8	eno	enolase 1/2/3	up	6.45
A0A087B4R0	tktAB	Transketolase	up	6.19
A0A0L0LVN5	gatC	Aspartyl/glutamyl-tRNA (Asn/Gln) amidotransferase subunit C	up	6.04
A0A068Z383	MutT	DNA mismatch repair protein	up	6.01
A0A1C7FV15	gadC	Amino acid permease	up	6.01
A0A087AF24	IF2	Translation initiation factor IF-2	up	5.52
D3R5K0	dapE	Conserved membrane protein (succinyl-diaminopimelate desuccinylase)	up	4.77
K4ILB4	talB	Transaldolase	up	4.23
A0A086ZT91	-	Gp18	up	3.71
A0A315RUP5	MTH2	8-oxo-dGTP diphosphatase	up	3.50
D3R6Z0	-	Collagen adhesion protein	up	3.28
A0A087CFX6	rplX	50 S ribosomal protein L24	up	3.23
A0A0L7BVT2	rpmG	50 S ribosomal protein L33	up	3.17
D3R5P9	livM	ABC transporter permease protein	up	3.03
A0A2M9H761	gapA	Type I glyceraldehyde-3-phosphate dehydrogenase	up	2.80
A0A315S119	nrdH	NrdH-redoxin	up	2.77
A0A0L7BLN2	-	DeoR family transcriptional regulator	up	2.55
A0A0L7BTZ0	-	Uncharacterized protein	up	2.51
A0A087DR62	infA	Translation initiation factor IF-1	up	2.44
A0A0L7BN57	priA	Phosphoribosylanthranilate isomerase PriA	up	2.34
A0A261G534	fadD	AMP-binding protein	up	2.33
A0A0L7BLU9	efp	Elongation factor P	up	2.31
B8DWS1	atpC	ATP synthase epsilon chain	up	2.28
A0A315RWG9	ctpA	Copper-translocating P-type ATPase	up	2.26
A0A2N3QGE9	ffh	Signal recognition particle protein	up	2.18
A0A2N3QFA6	rplR	50 S ribosomal protein L18	up	2.10
B8DV28	-	Zn-ribbon protein	up	2.07
A0A2N5J2G0	-	Argininosuccinate synthase	up	2.05
A0A315S096	fadD	Long-chain fatty acid-CoA ligase	up	2.03
D3R3Q3	-	Hypothetical transporter (Sulfate transport family)	up	2.02
A0A315S6K7	purC	Phosphoribosylaminoimidazole-succinocarboxamide synthase	up	2.01
A0A086ZTE6	talA	Transaldolase	down	0.50
A0A315RY65	sufC	Fe-S cluster assembly ATPase SufC	down	0.49
D3R515	yajC	YajC	down	0.49
A0A1C7FUB9	ftsH	ATP-dependent zinc metalloprotease FtsH	down	0.49
D3R4Q0	malL	oligo-1,6-glucosidase	down	0.49
D3R3A3	ppk1	Polyphosphate kinase	down	0.49
A0A1V8PLA3	ahpC	Alkyl hydroperoxide reductase	down	0.49
D3R6J3	MalT	MalT regulatory protein	down	0.49
D3R4I6	-	DedA family protein	down	0.48
B8DSM1	musE	Possible solute binding protein of ABC transporter	down	0.48
A0A261G6F6	ACSL	AMP-binding protein	down	0.48
A0A0L7BT82	-	ABC transporter permease	down	0.47
A0A0L7CYV9	hupB	Integration host factor	down	0.46
A0A386K0D5	musE	ABC transporter substrate-binding protein	down	0.46
A0A133L2E1	xfp	D-xylulose 5-phosphate/D-fructose 6-phosphate phosphoketolase	down	0.46
D3R7L4	srtA	Putative integral membrane protein	down	0.46
B8DW33	secY	Protein translocase subunit SecY	down	0.45
A0A0L7BMA7	aqpZ	Major intrinsic protein	down	0.45
A0A315T437	K06890	BAX inhibitor (BI)-1/YccA family protein	down	0.44
A0A087DDX0	srtA	Sortase	down	0.44
D3R4S6	butAC	(S,S)-butane-2,3-diol dehydrogenase	down	0.43
D3R7N6	-	Transporter	down	0.43
A0A0L7BSH9	-	ABC transporter substrate-binding protein	down	0.42
A0A087CM59	rpmA	50 S ribosomal protein L27	down	0.41
D3R7X1	htsT	Membrane-bound protein	down	0.39
D3R462	xynB	Beta-xylosidase	down	0.37
A0A087D495	mrp	Iron-sulfur cluster carrier protein	down	0.36
D3R5S2	pflACE	Pyruvate formate-lyase activating enzyme	down	0.36
A0A133L2D5	pflD	Formate C-acetyltransferase	down	0.35
A0A0A0UBM4	trxA	Thioredoxin	down	0.35
A0A261FCR3	ftsH	ATP-dependent zinc metalloprotease FtsH	down	0.34
A0A2T3G7W9	livK	ABC transporter substrate-binding protein	down	0.34
Q564C5	galR	LacI family DNA-binding transcriptional regulator	down	0.33
D3R5W5	amdH	Dihydrodipicolinate reductase	down	0.27
D3R5W3	-	GTP pyrophosphokinase	down	0.23
A0A315T5L1	mntH	Divalent metal cation transporter MntH	down	0.19
A0A174A5I1	pflD	Formate acetyltransferase	down	0.18
A0A126SV27	hupB	Integration host factor	down	0.17

**Table 3:** The potential oxygen-resistant genes identified in *B. animalis* AR668-R1.

The ID in Database	Gene ID	Gene	Predict Functions
D3R5K0	*gene0321*	*dapE*	membrane protein
A0A315RUP5	*gene0618*	*mth2*	7,8-dihydro-8-oxoguanine-triphosphatase
A0A068Z383	*gene0924*	*mutT*	DNA mismatch repair protein MutT
D3R6Y8	*gene1072*	*eno*	enolase
D3R4Y3	*gene1102*	*msrAB*	peptide methionine sulfoxide reductase MsrA/MsrB

## Data Availability

The original contributions presented in this study are included in the article/Supplementary Materials. Further inquiries can be directed to the corresponding author.

## Supplementary Materials

The following supporting information can be downloaded at: https://www.mdpi.com/article/10.3390/foods14101766/s1, Table S1. The DNA sequences of potential oxygen tolerance genes; Table S2. Primers used in this study; Table S3. Hydrogen peroxide tolerance of *Bifidobacterium*; Figure S1. Oxygen-tolerant *B. animalis* AR668-R1 obtained through ALE; Figure S2. Electrophoresis analysis for validation of plasmid construction. The PCR validation results of colonies 1–5 are *gene0321*, *gene0618*, gene0924, *gene1072*, and *gene1102*, respectively. M: DL2000 DNA Marker; Figure S3. Transcriptional analysis of oxygen-tolerance related genes in engineered *B. animalis* AR668-R1.

## References

[1] Evdokimova S.A., Karetkin B.A., Guseva E.V., Gordienko M.G., Khabibulina N.V., Panfilov V.I., Menshutina N.V., Gradova N.B. (2022). A study and modeling of *Bifidobacterium* and *Bacillus* coculture continuous fermentation under distal intestine simulated conditions. Microorganisms.

[2] Ji J., Lin W., Liu S.J., Jiao Z., Li X. (2023). Probiotics, prebiotics, and postbiotics in health and disease. MedComm.

[3] Sun Y., Guo S., Kwok L.Y., Sun Z., Wang J., Zhang H. (2024). Probiotic *Bifidobacterium animalis* ssp. *lactis* Probio-M8 improves the fermentation and probiotic properties of fermented milk. J. Dairy Sci..

[4] Zhang Y., Hou Y., Zhang S., Jing N., Zhang H., Xie Y., Liu H., Yan J., Ren J., Jin J. (2023). *Bifidobacterium animalis* A12, a probiotic strain that promotes glucose and lipid metabolism, improved the texture and aroma of the fermented sausage. Foods.

[5] Pimentel T.C., de Assis B.B.T., dos Santos Rocha C., Marcolino V.A., Rosset M., Magnani M. (2022). Prebiotics in non-dairy products: Technological and physiological functionality, challenges, and perspectives. Food Biosci..

[6] Baussier C., Oriol C., Durand S., Py B., Mandin P. (2024). Small RNA OxyS induces resistance to aminoglycosides during oxidative stress by controlling Fe–S cluster biogenesis in *Escherichia coli*. Proc. Natl. Acad. Sci. USA.

[7] Cronin M., Zomer A., Fitzgerald G., van Sinderen D. (2012). Identification of iron-regulated genes of *Bifidobacterium breve* UCC2003 as a basis for controlled gene expression. Bioengineered.

[8] Schöpping M., Vesth T., Jensen K., Franzén C.J., Zeidan A.A. (2022). Genome-wide assessment of stress-associated genes in bifidobacteria. Appl. Environ. Microbiol..

[9] Zomer A., van Sinderen D. (2010). Intertwinement of stress response regulons in *Bifidobacterium breve* UCC2003. Gut Microbes.

[10] Satoh T., Todoroki M., Kobayashi K., Niimura Y., Kawasaki S. (2019). Purified thioredoxin reductase from O_2_-sensitive *Bifidobacterium bifidum* degrades H_2_O_2_ by interacting with alkyl hydroperoxide reductase. Anaerobe.

[11] Schöpping M., Zeidan A.A., Franzén C.J. (2022). Stress response in bifidobacteria. Microbiol. Mol. Biol. Rev..

[12] Huang G., Pan H., Zhu Z., Li Q. (2020). The complete genome sequence of *Bifidobacterium longum* LTBL16, a potential probiotic strain from healthy centenarians with strong antioxidant activity. Genomics.

[13] Nguyen A.V., Yaghoobi M., Zhang S., Li P., Li Q., Dogan B., Ahnrud G.P., Flock G., Marek P., Simpson K.W. (2024). Adaptive Laboratory Evolution of Probiotics toward Oxidative Stress Using a Microfluidic-Based Platform. Small.

[14] Liu Y., Zhong W., Feng S., Tang Z., Zhang Y., Ai L., Xiong Z. (2023). Identification of new reference genes for colony counting by reverse-transcription quantitative PCR in *Bifidobacterium animalis*. J. Dairy Sci..

[15] Zhang D., Yang M., Song X., Xia Y., Ai L., Xiong Z. (2025). Oxygen tolerance mechanism of *Bifidobacterium animalis* AR668-R1 based on genomic and phenotypic analyses. LWT.

[16] Sahoo S., Mahapatra S.R., Misra N., Suar M., Panda S.K., Kellershohn J., Russell I. (2021). Application of genomics, transcriptomics, and proteomics in probiotic research. Probiotic Beverages.

[17] Li J., Xiong Z., Yang M., Song X., Wang G., Xia Y., Ai L. (2024). Improvement of electroporation-mediated transformation efficiency for *Bifidobacterium animalis* AR668. Food Biosci..

[18] Villa-Rodríguez E., Ibarra-Gámez C., de Los Santos-Villalobos S. (2018). Extraction of high-quality RNA from *Bacillus subtilis* with a lysozyme pre-treatment followed by the Trizol method. J. Microbiol. Methods.

[19] Kocaefe-Özşen N., Yilmaz B., Alkım C., Arslan M., Topaloğlu A., Kısakesen H.L., Gülsev E., Çakar Z.P. (2022). Physiological and molecular characterization of an oxidative stress-resistant *Saccharomyces cerevisiae* strain obtained by evolutionary engineering. Front. Microbiol..

[20] Starosta A.L., Lassak J., Jung K., Wilson D.N. (2014). The bacterial translation stress response. FEMS Microbiol. Rev..

[21] Gao X., Kong J., Zhu H., Mao B., Cui S., Zhao J. (2022). *Lactobacillus*, *Bifidobacterium* and *Lactococcus* response to environmental stress: Mechanisms and application of cross-protection to improve resistance against freeze-drying. J. Appl. Microbiol..

[22] Zafar H., Saier M.H. (2022). Comparative analyses of the transport proteins encoded within the genomes of nine *Bifidobacterium* species. Microb. Physiol..

[23] Duboux S., Muller J.A., De Franceschi F., Mercenier A., Kleerebezem M. (2022). Using fluorescent promoter-reporters to study sugar utilization control in *Bifidobacterium longum* NCC 2705. Sci. Rep..

[24] Schöpping M., Goel A., Jensen K., Faria R.A., Franzén C.J., Zeidan A.A. (2023). Novel Insights into the molecular mechanisms underlying robustness and stability in probiotic bifidobacteria. Appl. Environ. Microbiol..

[25] Zuo F., Yu R., Xiao M., Khaskheli G., Sun X., Ma H., Ren F., Zhang B., Chen S. (2018). Transcriptomic analysis of *Bifidobacterium longum* subsp. *longum* BBMN68 in response to oxidative shock. Sci. Rep..

[26] Zhu M., Dai X. (2019). Maintenance of translational elongation rate underlies the survival of *Escherichia coli* during oxidative stress. Nucleic Acids Res..

[27] Ha K.P., Edwards A.M. (2021). DNA repair in *Staphylococcus aureus*. Microbiol. Mol. Biol. Rev..

[28] Zhang B., Xu J., Sun M., Yu P., Ma Y., Xie L., Chen L. (2023). Comparative secretomic and proteomic analysis reveal multiple defensive strategies developed by *Vibrio cholerae* against the heavy metal (Cd^2+^, Ni^2+^, Pb^2+^, and Zn^2+^) stresses. Front. Microbiol..

[29] Scheible M., Nguyen C.T., Luong T.T., Lee J.H., Chen Y.W., Chang C., Wittchen M., Camacho M.I., Tiner B.L., Wu C. (2022). The fused methionine sulfoxide reductase MsrAB promotes oxidative stress defense and bacterial virulence in *Fusobacterium nucleatum*. MBio.

