# Supplementary material for: Proteomic Analysis of Bifidobacterium animalis AR668 and AR668-R1 Under Aerobic Culture
Source: Foods. 2025 May 16;14(10):1766. doi: 10.3390/foods14101766 (PMC12110750; doi:10.3390/foods14101766)

Table S1 The DNA sequences of potential oxygen tolerance genes

Gene	DNA sequence
gene0321	ATGGGCGGCCTCATCAGCGCAATGGAGGGATTCTGCGTCATCGGCATCGTGATCGCGGCC<br>GGCTATGCGGCCGCGCGTCTGCAGATCGGCGGCGCCCAGGCACAGTATGTGTTCAATCGC<br>CTGAGCTTCTTCATTGCGAGCCCGTGCCTCATGTTCGCCATTCTGGCGCAGGAGAACATCA<br>CACATCTGTTCGATTCCACGATCATCGTCGCGTTCTGCTCGGCGGTCGGCGTCGGACTCGT<br>CTTCCTCGTGCTCAACAGATTGTTCTTCCACCTCAAGGCACCGGATGCCACGATCGGCGTG<br>CTTAACTCGCTGTATTTGAATTCGAACAACATCGGCCTGCCCATCGCCACGTACATTCTCGG<br>CAACCCGGCGCTCGTCGCGCCCATTCTCGTCATGCAGCAGGCGCTGTTCACGCCGGTCGG<br>ACTCACCGTGCTCGACGTGACCACCAAGGGCAAGTTCTCGGTGAAGCAGGTGCTCAAGC<br>AGCCGTTGCACCAGCCATTGCTCATCGGCACCGTGCTCGGCATCATCGTCTCGGTCGTCTC<br>GTCGAAGGTGGGCCATTTCATTGTGCCGAACTTCCTGTTCGACCCGATCGACATGATTGGA<br>GATTCCGCGGTGCCGATGATCCTCATGGCATTCGGCATGTCGCTGCACGGCACGAAGCCG<br>ATGCAGAACAAGTCGAATCTGCCGGCGATCTGGACGGTGGCGGCGCTCAAGAACATCGT<br>GATGCCGCTCATCGCATTCGGCATCGCCTTCGCCATGGGCTTCCGCGGGCCCACGCTGTAC<br>GGCTGTGTCGTGCTCGCCGCACTGCCCACCGGCCAGAACGTGTACAACTACGCGGCCCGC<br>TACAATGTCGGCATGACCTTCGCGCGCTACGGCATACTCATCTCCACGATGACGAGCCCCAT<br>CGTCATCGCGATCATCGCCGCGCTCCTCAGCTGA
gene0618	ATGGAAGAGCCGAAACTGATTAAAGTGGTCGGAGCCGCCATCATCAAAGATGGCAAGGT<br>GCTGTGCGCGCAACGCGGCGCAGGCAAATCTCTCGCCGGGTATTGGGAGTTTCCCGGTG<br>GCAAGATCGAAGCGGGCGAGACACCTCAGCAGGCGCTCCAGCGGGAGATCGAGGAGGA<br>GCTGCTCTGCGAGATTGACATCGACAAGAAGGTGTGCACCAGCGACTACCTCTACGATTTC<br>GGCAACGTGCAACTCACCACCTTCCTGTGCCATCTCATCGAGGGAATGCCCAGACTTACG<br>GAACATGAGTGCATCGAATGGGTGGAGCCTTCGCAGATGCCCCGTCTGCGCTGGGCGCC<br>AGTGGATCATGATGCCGTGACGCGAATCTCCGGCATGGCATTCTGA
gene0924	ATGACTGAGGAACCATTGAGTCTTGAGCTCGAAGATGCCGAATGGCCGTTCGAATATATTG

	ATCATGATCGGCCAATCGCGCGGGCCATCGTCATCGATGCTCACGCGTACTTCTATTTTCTG
	CGGGTGCAGCGTACCGACGTCTTCGGCAATGCGGTGACGATCGAAACCTCCGGAGGCGG
	CATCGAACCAGGTGAGACTCCCACGGAGGCGGTGATGCGCGAGGTTAGGGAGGAGCTT
	GGAGCTGAGATTGAGATTCTCGGCGAGATTGGCTTGGTCAGCGATTGCTATAATCGGATTC
	ACCGCCATAATATCAATCACTACTACCTGTGCCAGGCGCTTTCGTTCGGCGAGACGAATCT
	GACGGAGCTGGAGCGGACGAGATTCAATCTGTCGACATTGAAGCTCACCTATGCTCAGGC
	CATCGCCGAATACAGGAGATGCGAGGATTCCAAGCTCGGCAGACTGATTGCGGCGCGTG
	AGCTGCCAATGCTGGAGCGAGCATGGGGCATGCTCGGAAAGTTGGGATATTAG
	GTGGCAGCAATTGAAAGCGTGTACGCACGCCAGATTCTTGATTCCCGCGGCAACCCGACC
	GTTGAGGTCGTGCTCGACACCGATGACGGTGCTCGCGGCCTGGGTCTCGTTCCCTCCGGC
	GCCTCCACCGGCGAAGCCGAGGCTTGGGAGCGTCGCGATGGCGACAAGTCCGTCTATCA
	GGGCAAGGGTGTCCTCGGTGCCGTCAAGGCGGTGAACGAGGAGATCGCCCCGAAGGTC
	ATCGGCATGGACGCCACCGACCAGCGCGCACTCGATGACCTGATGATCGAGCTCGACGGC
	ACCCCGAACAAGGGCCGTCTGGGCGCCAACGCCATCCTCGGTGTCTCCCTCGCCGCCCTT
	TATGCGGCCGCCGAGAGCGCCGAACTGCCGCTGTACCGCTATATCGGCGGCACCAACGGC
	CATGTTCTCCCGGTTCCGAACATGAACATCATGAACGGCGGCGCACACGCTGACTTCGCC
	ACCGACATCCAGGAGTACATGATCTCCCCGTACGGCTTCCAGACCTACTCCGAGGCTCTGC
gene1072	AGGCCGGCGTCGAGGTGTACCACACCCTCAAGAACGTGCTGAAGAAGCAGGGTCTGGCC
	ACCGGTCTCGGCGATGAGGGCGGCTTCGCTCCGAAGATGAAGACCAACGAGGATTCCCT
	CAAGTACATCATGGACGCCATCTCCGCCGCCGGCTATGAGCCGGGCAAGCAGATCGGCAT
	CGCCCTCGATGTCGCCTCCTCCGAGTTCTACAACAAGGAGACCGGCAAGTACCACTTCGAT
	GGTGAGGACCGTGATTCCGAGTACATGCTCGACTTCTACGAGAAGCTCGTCGACCAGTTC
	CCGATCGTCTCCATCGAGGATCCGTTCCAGGAGGAAGGCTGGGAAGACTGGGCCAAGAT
	CACCAAGGCCCTTGGCGACCGCCTGCAGTTCGTCGGCGACGATCTGTTCGTCACCAACCC
	GGTGCGTCTGAAGAAGGGCATCGATATGGGCGCCGGCAACTCCCTGCTGGTCAAGCTCA
	ACCAGATTGGCACCGTTTCCGAGACCCTCGACGCCATCGAGCTGGCCACGAAGAACGGC
	TTCACCTCCATGGTCTCCCATCGTTCGGGCGAGACGCCGGACACCACGATCTCCGATCTCG

	CCGTCGCCAAGAACACCGGCCAGATCAAGACCGGCGCCCCGGCACGTGGCGAGCGCATC
	GCCAAGTACAACCGCCTGCTCGAGATTGAGGAAGAGCTCGGTTCGACCGCCGAATACGCT
	GGCTACAGCGCATTCAAGGCATGCCGGAAGTACATGTGA
	ATGCAACGGACGGAGGCGGCCGCCCCCGAGACGCATGTGGCCTATATGGCCGGCGGATG
	CTTCTGGGGACTCGAACGAGCCATGCAGAATGTCGATGGCGTCCTTGACACCACGGTGG
	GATACGCGCAATCCATTACGCCGGATCCCACCTATCGCATGGTGTGTTCGGGAACGACCCA
	CGCCGTGGAAACGGTGCGCATCGATTACAATCCGACGCGGGTGAGTCTGCGAACACTGA
	CCTTGCTGTTCCTGAGCATCATCGATCCATTCAGCGTCGACCGTCAGGGCAACGACGTCGG
	GTCGCAATACCGTTCCGGACTGTATCCGGCCGGTGCGCATGCCGCCGAGCAGCGCGCGGT
	GTATGAGCAGGCGTTGGACGAACTCGCGCAACGCTCCGGATCGACTCCGGCCGTTGAGA
	TCGAGGATTTGCGCAATTTCACGATTGCGGAGCCCGAACATCAGGATTACCTGCTCGCCAA
gene1102	TCCAACAGGCTACTGTCATATTCCACTGAGCGTGATCGACCACATGCGCGAACGTCAGCGT
	CATATCGAACGCATCTGGAGCCTGAGCCCGGAACAGTATGCGGTCACGCAGCAGTCTGCC
	ACCGAACCGCCGTTCCATAACGAATATGATCGTCTGTTCGACCCCGGCATCTATGTGGATCG
	AGTCAGCGGCGAACCGTTGTTCTTCTCGACCGACAAATTCGATTCCGGCTGTGGCTGGCC
	GTCGTTCAGCACGCCGATCGATCAGGGGTCGGTACGCATGGTCAAGGATTACAGCTTGCC
	GCTGCACCCGCGCCTCGAGGTGCGGGCCGTGCAATCCGACAGTCATCTGGGCCATGTGTT
	CGCAGACGGCCCCCGCGAACGTGGCGGCATGCGGTACTGCATGAATTCGGCGTCACTTCG
	ATTCGTACCGCGAGATCGGATGGCCGAGGAAGGCTATGGGGATTTGGTAGGAGAGCTCG
	ACGCGCGGCTGAGCGCTTCTGCCGGCGAGTGA

Table S2 Primers used in this study.

Primer	Sequence (5’ to 3’)	Description
qPCRgene0321-F	TCGTCTTCCTCGTGCTCAAC	For the transcription analysis of oxygen-tolerance genes
qPCRgene0321-R	AGAATGTACGTGGCGATGGG	
qPCRgene0618-F	ATTTCGGCAACGTGCAACTC	
qPCRgene0618-R	CTCCACCCATTCGATGCACT	
qPCRgene0924-F	CTTGAGCTCGAAGATGCCGA	
qPCRgene0924-R	GCTGCACCCGCAGAAAATAG	
qPCRgene1072-F	GAAGAACGGCTTCACCTCCA	
qPCRgene1072-R	CTTGATCTGGCCGGTGTTCT	
qPCRgene1102-F	CAGTCATCTGGGCCATGTGT	
qPCRgene1102-R	TCTCGCGGTACGAATCGAAG	
GBDgene0321-F	ATGGGCGGCCTCATCAG	For pLYP0321 construction
GBDgene0321-R	TCAGCTGAGGAGCGCGGC	
GBDgene0618-F	TCAGAATGCCATGCCGGA	For pLYP0618 construction
GBDgene0618-R	ATGGAAGAGCCGAAACTG	
GBDgene0924-F	CTAATATCCCAACTTTCC	For pLYP0924 construction
GBDgene0924-R	ATGACTGAGGAACCATTG	
GBDgene1072-F	GTGGCAGCAATTGAAAGC	For pLYP1072 construction
GBDgene1072-R	TCACATGTACTTCCGGCA	
GBDgene1102-F	ATGCAACGGACGGAGGCG	For pLYP1102 construction
GBDgene1102-R	TCACTCGCCGGCAGAAGC	

TY-pAM1ldh2-F	CGGAGTAGCAAGCGACGTGAT	For PCR verification of oxygen-tolerance genes
TY-pAM1ldh2-R	AAGAATCGCGAGAGGCACCG	

Table S3 Hydrogen peroxide tolerance of *Bifidobacterium*

	AR668			AR668-R1		
Concentration/ mM	3.5	4.5	6.0	8.5	10.0	12.0
Survival/ %	132.62±1.83	6.17±0.72	0	9.733±0.58	0.013±0.23	0

Figure S1 Oxygen-tolerant *B. animalis* AR668-R1 obtained through ALE

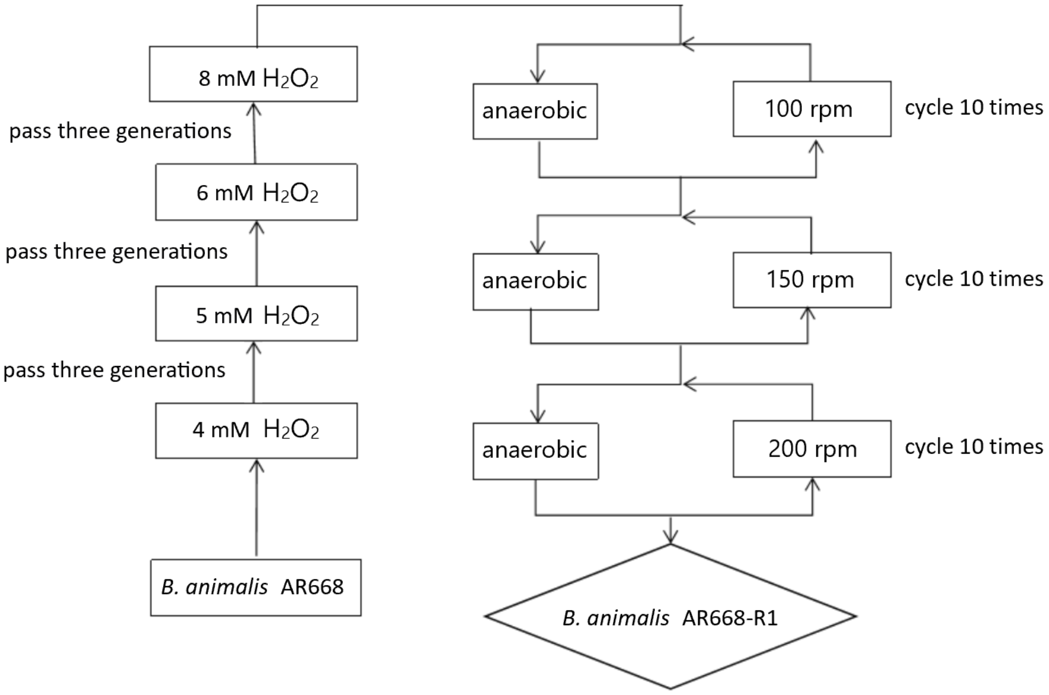

Figure S2 Electrophoresis analysis for validation of plasmid construction. The PCR validation results of colonies 1-5 are *gene0321*, *gene0618*, gene0924, *gene1072*, and *gene1102*, respectively. M: DL2000 DNA Marker.

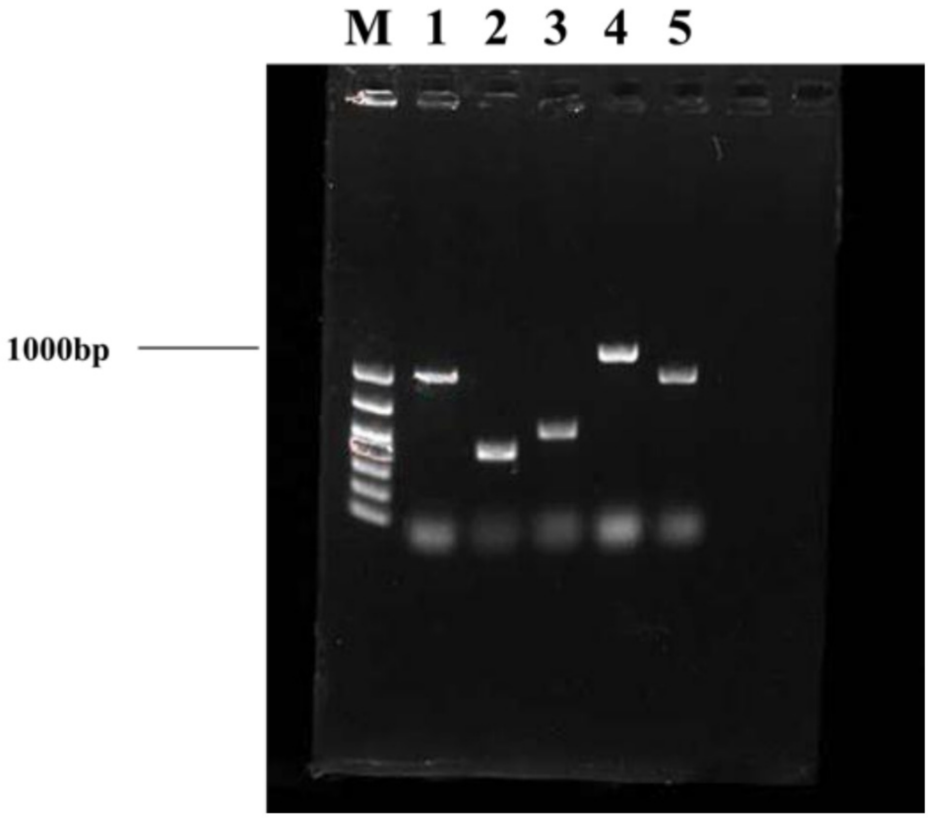

Figure S3 Transcriptional analysis of oxygen-tolerance related genes in engineered *B. animalis* AR668-R1.

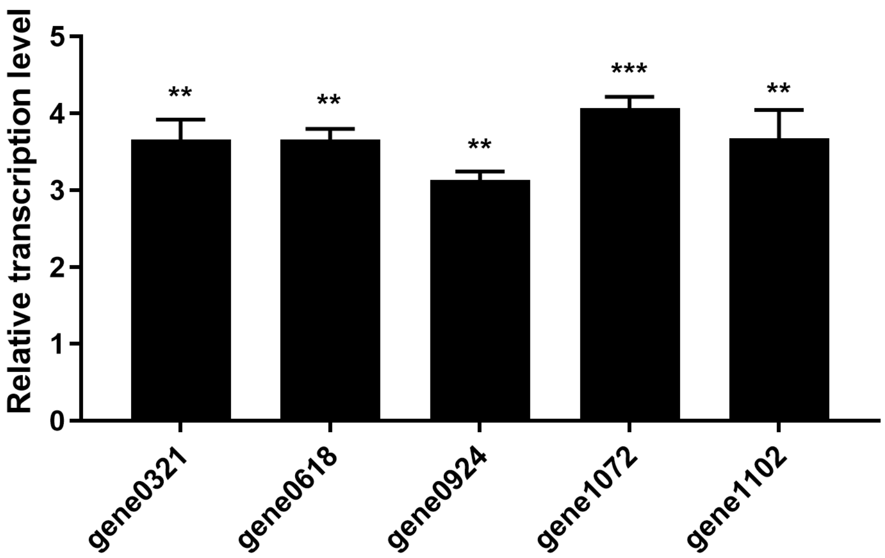

Supplement: Supplementary file 1 [file foods-14-01766-s001.zip › foods-3577937-supplementary.pdf]
